# Management Strategies to Mitigate N_2_O Emissions in Agriculture

**DOI:** 10.3390/life12030439

**Published:** 2022-03-17

**Authors:** Muhammad Umair Hassan, Muhammad Aamer, Athar Mahmood, Masood Iqbal Awan, Lorenzo Barbanti, Mahmoud F. Seleiman, Ghous Bakhsh, Hiba M. Alkharabsheh, Emre Babur, Jinhua Shao, Adnan Rasheed, Guoqin Huang

**Affiliations:** 1 Research Center on Ecological Sciences, Jiangxi Agricultural University, Nanchang 330045, China; muhassanuaf@gmail.com (M.U.H.); muhammadaamer@jxau.edu.cn (M.A.); jinhuashao1@gmail.com (J.S.); 2 Department of Agronomy, University of Agriculture, Faisalabad 38040, Pakistan; athar.mahmood@uaf.edu.pk; 3 Department of Agronomy, Sub-Campus Depalpur, Okara, University of Agriculture, Faisalabad 38040, Pakistan; masood.awan@uaf.edu.pk; 4 Department of Agriculture, Food Sciences University of Bologna, 40127 Bologna, Italy; lorenzo.barbanti@unibo.it; 5 Plant Production Department, College of Food and Agriculture Sciences, King Saud University, Riyadh 11451, Saudi Arabia; mseleiman@ksu.edu.sa; 6 Department of Crop Sciences, Faculty of Agriculture, Menoufia University, Shibin El-Kom 32514, Egypt; 7 Training and Publicity, Agriculture Extension, Dera Allah Yar 79000, Pakistan; ghouskhosa2353@gmail.com; 8 Department of Water Resources and Environmental Management, Faculty of Agricultural Technology, Al Balqa Applied University, Salt 19117, Jordan; drhibakh@bau.edu.jo; 9 Department of Forest Engineering, Faculty of Forestry, Kahramanmaras Sutcu Imam University, Kahramanmaras 46050, Turkey; emrebabur@ksu.edu.tr; 10 Key Laboratory of Crops Physiology, Ecology and Genetic Breeding, Ministry of Education/College of Agronomy, Jiangxi Agricultural University, Nanchang 330045, China; adnanbreeder@yahoo.com

**Keywords:** N_2_O emissions, denitrification, nitrification, C:N ratio, integrated nutrient management

## Abstract

The concentration of greenhouse gases (GHGs) in the atmosphere has been increasing since the beginning of the industrial revolution. Nitrous oxide (N_2_O) is one of the mightiest GHGs, and agriculture is one of the main sources of N_2_O emissions. In this paper, we reviewed the mechanisms triggering N_2_O emissions and the role of agricultural practices in their mitigation. The amount of N_2_O produced from the soil through the combined processes of nitrification and denitrification is profoundly influenced by temperature, moisture, carbon, nitrogen and oxygen contents. These factors can be manipulated to a significant extent through field management practices, influencing N_2_O emission. The relationships between N_2_O occurrence and factors regulating it are an important premise for devising mitigation strategies. Here, we evaluated various options in the literature and found that N_2_O emissions can be effectively reduced by intervening on time and through the method of N supply (30–40%, with peaks up to 80%), tillage and irrigation practices (both in non-univocal way), use of amendments, such as biochar and lime (up to 80%), use of slow-release fertilizers and/or nitrification inhibitors (up to 50%), plant treatment with arbuscular mycorrhizal fungi (up to 75%), appropriate crop rotations and schemes (up to 50%), and integrated nutrient management (in a non-univocal way). In conclusion, acting on N supply (fertilizer type, dose, time, method, etc.) is the most straightforward way to achieve significant N_2_O reductions without compromising crop yields. However, tuning the rest of crop management (tillage, irrigation, rotation, etc.) to principles of good agricultural practices is also advisable, as it can fetch significant N_2_O abatement vs. the risk of unexpected rise, which can be incurred by unwary management.

## 1. Introduction

The sustainability of agricultural activities involves supporting crop yields under adverse natural conditions [1,2,3,4,5,6,7,8,9]. Many countries across the globe have adopted intensive agricultural practices to assure food security under the rapid increase in world population [10,11]. However, scaling up the level of crop intensiveness has devastating impacts on the environment [12]. Agriculture is a major contributor to greenhouse gases (GHGs) (namely, CO_2_, N_2_O and CH_4_) released into the atmosphere and accounts for 10–12% of the total GHGs produced globally by anthropogenic activities [13,14]. These GHGs are a major source of global warming and climate change across the globe and pose a serious threat to global food security [15,16].

N_2_O is a powerful and long-lasting GHG, has a global warming potential (GWP) 298 times as high as that of CO_2_ and can contribute to the depletion of the stratospheric ozone layer [17]. Moreover, it is a very reactive gas, which catalyzes the production of the tropospheric ozone, exerting adverse impacts on humans and crop production [18,19]. Agriculture is responsible for about 60% of the global N_2_O production, owing to the heavy usage of mineral N and the sustained use of legumes as cover and main crops releasing N at the end of their life cycle [20,21,22]. For example, from 1990 to 2005, agricultural emissions have increased by 14%, with an average increase of 49 Mt CO_2_ per year [23]. Based on another source, during the last decade, approximately 80% of the world’s total N_2_O emissions were related to agricultural activities, with the concentration in atmosphere increasing from 270 ppb to 319 ppb [24]. Moreover, N_2_O emissions are expected to increase by 35–60% in the near future, largely due to poor manure management and increased application of chemical fertilizers [24]. Additionally, excessive use and inappropriate timing of N application can lead to N leaching that affects water quality [25], resulting in increased N_2_O emission from the landscape-draining waterways [26]. 

**Figure 1:**
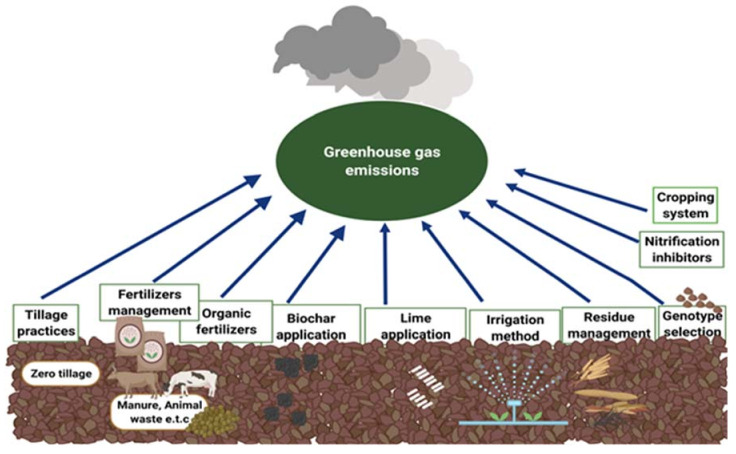
Management practices influencing N_2_O emissions to the atmosphere. The adoption of several measures in each specific management sector can contribute to mitigate N_2_O emission from agricultural soils.

In soils, N_2_O is mainly produced by transformation of reactive N through the microbes [25,26,27,28,29]. When N enters the soil, either from organic or mineral fertilizers in the form of NH_4_^+^ and NO_3_^−^, there are different processes that can result in N_2_O formation. However, their relative prominence is still not well understood [30,31]. Three main processes, namely nitrification, denitrification and dissimilatory nitrate reductions, are considered the main contributors to N_2_O emissions [27]. The contribution of each process to N_2_O emission depends upon soil texture, organic C, soil pH, microbial activities and environmental conditions, including precipitation and temperature [28]. The quality and intricacy of N_2_O production pathways, and their spatial as well as temporal variability, make the reduction in N_2_O from soils quite challenging to interpret [32]. Crop management practices, including tillage and irrigation, N fertilizers, biochar, lime, nitrification inhibitors, slow-releasing fertilizers, arbuscular mycorrhizal fungi (AMF), suitable cultivars, appropriate crop rotations and integrated nutrient management (INM) can significantly influence soil properties, which in turn affect N_2_O emissions [33,34,35,36,37,38,39]. Therefore, it is generally sensed that emissions can be mitigated by the suitable management of tillage and irrigation practices, reducing the overall N application and using biochar, lime, organic amendments, manures, nitrification inhibitors, fermented fertilizers, AMF, suitable crop rotations and INM (Figure 1). 

To better appreciate the extent of these effects, organize in a comprehensive way the multiple contributions on this topic and discuss the variable results obtained in the quest to curb N_2_O emission, we set out to review the potential of different management options to reduce N_2_O emission on the basis of the available data. It is generally acknowledged that the adoption of suitable practices can play a significant role in restraining N_2_O emission, but the extent to which the atmospheric equilibrium and agricultural production will benefit from these efforts is still questioned.

## 2. N_2_O Production and Emission

Nitrous oxide is produced in the process of nitrification, consisting of the microbial conversion of ammonia (NH_3_) to nitrate (NO_3_^−^). Nitrification (NF) is considered the main process involved in the global N cycle. Most of the transformation of N during nitrification is mediated by autotrophic micro-organisms. The first step in nitrification is NH_3_ oxidation to the hydroxylamine (NH_2_OH). Both ammonia-oxidizing archaea (AOA) and ammonia-oxidizing bacteria (AOB) mediate this process. 

In various soils, the quantity of AOA is higher than AOB, which supports the hypothesis that the abundance of AOA can better control nitrification rates, in turn leading to lower N_2_O emission compared to soils with higher AOB [40,41]. This is especially true in the acidic soils, where AOA prevail as a result of their unique adaptation [42]. Nonetheless, the degree to which AOA vs. AOB can affect N_2_O emission is still uncertain [43] and might depend on the NH_2_OH fate. The metabolic and enzymatic pathways lead to decomposition of NH_2_OH into NO_2_^−^ and nitrogen oxide (NO) [44]. NO_2_^−^ is further volatilized into HONO, but NO_2_^−^ may be converted into NO, N_2_O and N_2_ via nitrifier denitrification [45,46]. 

In contrast to nitrification, denitrification (DNF) is a reduction process involved in the conversion of NO_3_^−^ to N_2_, mediated by facultative anaerobic bacteria [47]. This process can be completed up to N_2_ production, but if it remains incomplete, it results in N release in the form of NO and N_2_O [48].

The microbial processes of NF and DNF are responsible for 70% of global N_2_O emission [49,50]. However, the above description of the two processes as sources of N_2_O is a simplification, owing to the fact that the main process pathway can provide a wealth of collateral processes that either form or use N_2_O. Moreover, other metabolic processes can contribute to N_2_O production in soils:

- The decomposition of hydroxylamine during the process of autotrophic as well as heterotrophic nitrification;
- The chemical DNF of soil NO_2_^−^ and abiotic decomposition of ammonium nitrate in the presence of light, humidity and reacting surfaces;
- The production of N_2_O by nitrifier denitrification within the same nitrifying micro-organisms;
- The coupled nitrification–denitrification by different micro-organisms (the nitrite oxidizers produce nitrate, which is denitrified by denitrifiers in situ);
- The DNF conducted by microbes capable of using nitrogen oxides as alternative electron acceptors under O_2_ limited conditions;
- The co-denitrification of organic N compounds with NO and nitrate ammonification or dissimilatory nitrate reduction to ammonium [51].

## 3. Environmental and Anthropic Factors Affecting N_2_O Emission from Agricultural Soils

### 3.1. Soil pH 

**Figure 2:**
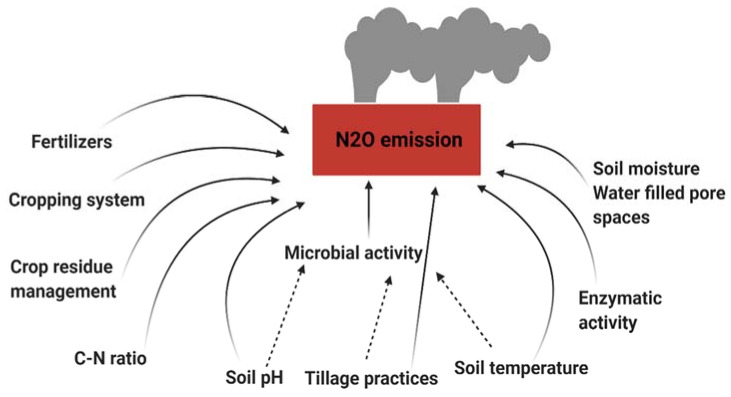
Factors and management practices responsible for N_2_O emission from agricultural soils.

Soil pH is one of the main factors that can affect N_2_O emission (Figure 2). The increase in soil pH can reduce the emission of N_2_O [52,53], although some other source reports increased N_2_O emission at increasing pH [54], which is consistent with denitrifying bacteria thriving on relatively high pH for their activities. Alkaline pH is considered responsible for enhancing the rates of both NF and DNF processes [55,56]. In general, soil pH influences the microbial population and activity, which directly impact N_2_O emission [57].

### 3.2. Soil Moisture and Temperature 

Large quantities of N_2_O are produced under high water-filled pore space (WFPS), owing to the fact that soil moisture controls N_2_O emission through organic matter (OM) decomposition. Soil moisture can enhance organic C mineralization, which can control microbial metabolism and activities [58,59]. Thus, higher C stimulates the activities of micro-organisms by increasing substrate availability, which in turn increases N_2_O emission. Moist soils enhance N_2_O emission over long periods, owing to increased availability of C substrate for microbial activities. Moreover, no tillage (NT) can increase the WFPS compared to conventional tillage (CT), which can be a reason for increased N_2_O emission under NT conditions. Soil temperature interacts with moisture in regulating N_2_O production. Bacterial populations increase with increasing temperature up to a certain range (25–35 °C) [60,61], and the activities of both nitrifying and denitrifying bacteria are equally enhanced at higher soil temperatures [62]. 

### 3.3. Application of Crop Residues 

The addition of crop residues and straw provides a source of easily available C and N, henceforth, a potential source of N_2_O emission [63]. Nitrogen mineralized from crop residues is quite easily dispersed in the form of N_2_O [64]. The release of N and C from mineralization of crop residues largely depends on the C:N ratio of the specific residues [65]. The rate of DNF depends on the amount of C that is made easily available to the pool of denitrifying bacteria [66]. High N_2_O emission from loamy soil was observed following the incorporation of straw with low C:N ratio [65], while low N_2_O emission from sandy soil was noticed with the addition of cereal straw with higher C:N compared to vegetable residues with lower C:N [67]. Therefore, the characteristics of crop residues incorporated into the soil can be a significant factor in N_2_O emissions [68].

### 3.4. Nitrogen Application 

Before 1950, less than 50% of N_2_O emission was caused by N fertilizers in the agricultural sector. Nonetheless, most of the N_2_O emissions were linked to animal rearing and related activities [69]. However, with the increase in human population and food demand, increased application of N fertilizers was also needed. Agriculture is responsible for more than 60% of N_2_O emission [21,22]. Nitrogen fertilizers have high mobility in soil solution: after application, they enter the soil, undergoing diverse reactions resulting in N leaching, immobilization, volatilization and DNF [70]. Therefore, N fertilizers have significant impact on N_2_O emission, leading to differentiated emissions according to fertilizer type [71]. The method and timing of N application also have substantial impact on N_2_O emission [72]. Among the application methods, the N applied as side banding significantly reduced N_2_O emission compared to broadcasting [73]. Similarly, the time of N application is very crucial, and the selection of suitable timing can contribute to N loss reduction. The available ammonium (NH_4_^+^) and nitrate (NO_3_^−^) are major sources of N_2_O emission from soils [74], and N fertilizers, which more or less directly supply the two N forms, are largely implied in N_2_O production and emission [75,76,77]. The deep placement of fertilizers has been seen to substantially improve crop growth compared to shallow and surface placement [78]. Plant roots tend to proliferate around the fertilizer area; therefore, deep placement considerably increased root density, N and water uptake from deeper layers in various cereals [78,79]. Moreover, in deep placement, a thicker layer must be crossed by diffusing N_2_O, which prolongs the residence time and favors the ultimate reduction of N_2_O to N_2_ in the upper topsoil where no fertilizer N was placed [78], resulting in significant reduction in N_2_O emission [79]. 

### 3.5. Soil Micro-Organisms

An increase in soil depth considerably decreases microbial biomass and activity. Microbial occurrence is imperative for NO_3_^−^ and NO_2_^−^ reduction to NO, N_2_O or N_2_; this reaction is coupled with electron transport in the DNF process [77]. Denitrifying bacteria have the ability to reduce NO_3_^−^, NO_2_^−^ and NO under soil anaerobic conditions. They catch the energy from sunlight and organic or inorganic substrates, and are consequently known as phototroph, organotroph or lithotroph. Moreover, some enzymes, including ammonia monooxygenase, hydroxylamine oxidoreductase and nitrite oxidoreductase, are involved in the NF, and these enzymes either increase or decrease N_2_O emission by affecting the rate of NF [80]. In a similar way, other enzymes, including nitrate reductase, nitrite reductase, nitric oxide reductase and nitrous oxide reductase, are involved in the DNF process. The occurrence and amount of these enzymes remarkably influence DNF rate and, consequently, N_2_O soil emission [80]. The amount of soil organic carbon positively influences N_2_O production and emission [81], also in association with soil moisture [82]. In fact, soil organic C provides the substrate for microbial growth that is needed for both NF and DNF processes [83].

### 3.6. Soil Characteristics

Fine textured soils emit more N_2_O [84], owing to the fact that they have more capillary pores within soil aggregates compared to sandy soils [85]. The pores present in fine soils hold more water, leading to anaerobic conditions, which are maintained for a longer time, resulting in significant increase in N_2_O emission compared to sandy soils [86]. The DNF process is also considerably increased, as soil texture becomes finer and WFPS increase [85]. When WFPS decrease, the DNF process is slowed. In fact, it was reported that in clayey soils, N_2_O emission was considerably increased with increasing WFPS, up to 40%, and reached its maximum extent at WFPS higher than 70% [85]. Generally, soil texture affects N_2_O emission by determining how likely it is for anaerobic vs. aerobic soil conditions to prevail [87,88]. Moreover, soil texture also affects N_2_O emission owing to differences in soil N availability, the amount of organic carbon and microbial population [89]. Site exposure influences soil temperature and moisture, in turn affecting N_2_O emission, as does field surface morphology; N_2_O emission was recorded maximum in depressions vs. ridges and sloped lands, owing to higher moisture content present in depressed areas [90,91]. Lastly, lower air pressure at high altitudes also favors higher N_2_O emissions due to a reduction in the counter pressure exerted on the soil [90,91]. 

## 4. Management Options to Mitigate N_2_O Emission 

### 4.1. Modification of Irrigation Pattern

Irrigation is an important factor in N_2_O emission [92]. The amount of water supplied and the method of distribution affect soil moisture spatially and temporally [93], and significantly impact on the N cycle. This includes the processes of NF and DNF on which N_2_O production depends [94,95]. 

Flood irrigation (FI) is the most common irrigation method in developing countries, such as India, Pakistan, Bangladesh and large parts of Africa. In FI, high volumes of water are applied to crops, resulting in fertilizers being strongly diluted and easily absorbed [94]. However, large irrigation volumes determine the anaerobic conditions conducive to N_2_O production and nitrate leaching [96]. To prevent this, a precise water application technique, such as alternate wetting and drying (AWD), could be useful to save water while concurrently reducing GHG emissions. However, contrasting results are reported about the effect of AWD on N_2_O emission and grain yields even in paddy rice, one of the crops most suited for AWD. On the one hand, Lahue et al. [97] found that AWD vs. FI curbed CH_4_ emission by 80% in a clay-loamy soil, while significantly increasing the final yield; on the other hand, Lagomarsino et al. [98] reported that AWD saved water by 70% and decreased CH_4_ emission by 97%, but it increased N_2_O emission by five times in a clayey soil.

Generally, AWD inhibits CH_4_ emission [77]; however, soil moisture during AWD cycles remains high, which can create anaerobic conditions [92] and favor N_2_O emission. Soils produce large quantities of N_2_O when WFPS fluctuates around 45–90% [99]. 

Under aerobic soil conditions, NF becomes the dominant N_2_O production pathway when WFPS increases up to 60–70% [100]. Conversely, DNF becomes a dominant pathway for N_2_O production when WFPS exceeds 60–70% [100]. However, the production of N_2_O may still be limited with WFPS around 50–60% as a result of dissimilatory nitrate reduction to ammonia [101]. For instance, continuous flooding in rice releases less N_2_O to the atmosphere [102,103], owing to water saturated conditions favoring ultimate NO_3_^−^ reduction to N_2_ by denitrifiers [51]. Conversely, AWD may be responsible for increased N_2_O emission when it determines soil cracks; stronger aeration at deeper layers increases NF and provides substrate for N_2_O emission [104,105]. 

Similarly, modifications in the irrigation method can play a crucial role in the amount of water used and N_2_O emission. Different patterns of water infiltration and redistribution result in variable time trends of soil water content and water infiltration depths; all this has a great impact on soil N_2_O emission and its spatial and temporal occurrence [106]. The surface layer in a field irrigated by sprinkler irrigation (SI) is relatively loose compared to FI. Therefore, in such soils, the NO_3_-N and NH_4_-N ions are less leached and remain more concentrated in the root zone [106,107], which makes them more easily absorbed by plant roots and, therefore, less prone to be turned into N_2_O [107,108]. SI is a water-saving approach, and soil conditions during SI, as well as drip irrigation (DI), favor NF in both cases. Enhanced NF provides the substrate for N_2_O emission [104,105,106]; however, SI is associated with modest WFPS, resulting more likely in reduced N_2_O emissions [109,110,111,112,113]. 

It is therefore evinced that more advanced irrigation methods, such as SI and DI, lead to a contained risk of N_2_O emission with respect to FI. A controversial role is played by AWD, which is proposed as an advanced version of FI: despite undeniable benefits in terms of water saving and crop performance, the unstable moisture conditions associated with AWD may be conducive to stronger N_2_O production. In general, it is sensed that the irrigation practice should be directed toward higher water use efficiency, either by replacing less efficient methods or better tuning the existing ones, as a premise for more balanced moisture conducive to less N_2_O emission.

### 4.2. Tillage Practices 

Tillage practices influence crop productivity [114] as well as GHG emission, as they substantially affect soil properties [115]. Tillage disturbs the soil and increases CO_2_ emission by aerating the soil and breaking soil aggregates, which release the organic carbon that favors microbial activities responsible for GHG emission [116].

It is not easy to univocally identify which tillage practices could reduce GHG emission [117], as contrasting results have been reported in the literature. In rice fields, Xiao et al. [118] and Liang et al. [119] noticed a substantial reduction in N_2_O under no tillage (NT) compared to conventional tillage (CT). Conversely, a meta-analysis conducted by Mei et al. [120], including rice and other arable crops (wheat, maize, others), showed that conservation tillage increases N_2_O emission by an average 17.8% compared to CT. Lastly, another meta-analysis conducted by Feng et al. [121] pointed out the advantage for NT in terms of N_2_O and CH_4_ reduction (−6.6% compared to CT). In this last source [121], special emphasis is given to the interactions of tillage with other crop management practices and land use patterns in triggering/mitigating GHG emission from agricultural soils. Despite the uncertainties in N_2_O effects, NT practices appreciably offset GHG emissions owing to C sequestration [122] and reduction in CH_4_ emissions. This results in the global warming potential (GWP) of NT being remarkably lower than that of CT [123,124]. In turn, this suggests that NT is beneficial for GHG emission and C-smart agriculture, and must be generally promoted in cropping systems. No tillage reduces the losses of OM and significantly increases soil bulk density (BD) [125]. The long-term use of NT can improve soil structure and reduce soil temperature, owing to the residues present on soil surface reflecting the incoming radiation and acting as a barrier between soil surface and atmospheric air [126]. This, in turn, may lead to reduced N_2_O emission compared to CT [127,128]. In another case, CT increased water-holding capacity, WFPS and the availability of substrate for microbial activities, potentially leading to increased N_2_O emission [129]. However, the effect of tillage practices can vary according to climate type. For instance, Van Kessel et al. [130] conducted a meta-analysis and found that dry warm climate significantly increases N_2_O emissions. Rainfall and temperature are considered key factors affecting N_2_O emissions. Higher rainfall increases soil moisture contents, which reduce the soil oxygen availability, which ultimately increases the NF and DNF and results in significant increase in N_2_O emissions. Therefore, in warm dry regions, NT can be an important practice to reduce N_2_O emission as compared to conventional tillage practices, which can increase N_2_O emission due to decomposition of organic matter and increase in microbial activities [131]. Recently, Shakoot et al. [132] (2022) also found that NT reduces N_2_O emissions in irrigated areas, whereas it increases N_2_O emission in rain-fed areas. 

The contrast among studies for N_2_O emissions could be ascribed to different soil characteristics, ambient conditions and time at which tillage practices are carried out in a specific soil. However, despite the non-univocal effects on N_2_O production, reduced and no tillage are associated with a beneficial effect, in general, in GHG mitigation. Therefore, as in the case of irrigation practices, it appears that more advanced tillage practices provide a more favorable background for the containment of GHG emission. 

### 4.3. Crop Residue Management

Crop residues (CR) return to the soils is widely popular, owing to its benefits in increasing agricultural production and soil fertility [133,134]. Moreover, CR return also influences N_2_O emissions by regulating the microbial activities, and C and N availability [135,136]. At a global level, it is estimated that CR return produces 0.4 million metric tons of N_2_O-N/year [137]. Nonetheless, contrasting results have been shown in the literature concerning the effects of CR return on N_2_O emission from agricultural soils, depending on several CR and soil characteristics. 

Various authors noted that returning CR can increase N_2_O emissions by increasing C and N availability for microbial activities and modifying soil aeration by improving soil aggregation and microbial demand, which is considered a major factor mediating soil NF and DNF for N_2_O production [126,135,138,139]. Conversely, other authors reported that the addition of CR has an inhibitory effect on N_2_O emission, depending on soil properties and C/N ratio of crop residues [140,141]. Additional soil characteristics influencing CR effects on N_2_O emission are soil pH, texture, water content and residue C and N input to soil [142,143,144]. Soil pH affects CR decomposition, and C and N availability for NF, as well as DNF [145]. Similarly, soil texture affects soil permeability and water conditions and, therefore, CR decomposition and N transformation processes [146]. Thus, it is important to consider the above-discussed soil and CR properties when estimating N_2_O emission from CR.

The return of CR can serve as a source of carbon for microbial growth, stimulating the N assimilation by micro-organisms. This action can prompt a strong competition for NH_4_^+^ between heterotrophic micro-organisms and autotrophic nitrifiers [147], resulting in N_2_O production. Additionally, CRs serve as source of energy for denitrifiers, enhancing DNF and, resultantly, N_2_O emission under aerobic conditions. In those agricultural systems where CRs are soil incorporated, they provide N and C for NF. For instance, coarse textured soils have low DNF owing to the limited availability of organic carbon [148,149], and the addition of CRs can result in increased N_2_O emission. Moreover, in fine textured soils, CR addition improves soil properties and increases substrate availability and microbial activities; therefore, the addition of CRs with low C:N ratio increases N_2_O emission from these soils [139]. Lastly, CRs from mature crops have higher C:N ratio and tend to immobilize N and reduce NO_3_^−^ availability, thus limiting N_2_O emission from agricultural soils [149]. The decomposition of CR interacts with soil water content in determining the O_2_ status in organic hotspots. For instance, CR significantly increased the N_2_O emission at 30 and 60% WFPS; however, after heavy rainfall and increase in WPFS at 90%, N_2_O emission was reduced by CR owing to a shift in N_2_O:N_2_ product ratio of DNF due to more reducing conditions [150]. Lastly, residue incorporation during the spring season following N addition of N fertilizers increases the potential interactions between external N source and decomposition of CR, which can increase the DNF and, subsequently, N_2_O emissions [151]. Even in CR management, it is perceived that no univocal behavior can be detected with respect to N_2_O emission. Several features, including CR characteristics and ambient conditions, must be considered to enhance smart CR management and its contribution to reduced N_2_O emission. The trade-offs for successful management are, nevertheless, undeniable.

### 4.4. Fertilizer Management 

#### 4.4.1. Adjusting Fertilizer Dose and Matching N Supply with Demand

The application of optimum levels of N and P fertilizers ensures higher yield and reduces background GHG emissions. N_2_O emissions from soils are influenced by fertilizer type, amount and application time [152]. The containment of N doses at the lowest non-limiting levels decreases the soil N availability and, consequently, the N_2_O emission [153]. 

Many experiments demonstrate a substantial increase in N_2_O emission with application of N fertilizers however, N_2_O emissions also varied according to source of N application (Table 1). In rice, N_2_O emission increased with an increase in N rate [154], which is supported by another experiment where a 33% reduction in the reference N application resulted in −28% N_2_O emissions [155]. In another study, it was noted that application of N (200 kg ha^−1^) reduced the methane emissions by 25–30% from rice crop as compared to application of N (400 kg ha^−1^) [156]. The N application method can also affect N_2_O production. In fact, N placement near the roots increased the nitrogen use efficiency (NUE) and reduced N_2_O emissions [157]. Moreover, optimizing N fertilizer use to better match nutrient supply with crop demand significantly reduced the soil amount of residual N, curbing N_2_O emission [158]. From a practical viewpoint, split fertilizer applications at different crop stages ensure uninterrupted N availability, which in turn improves NUE and reduces N_2_O emission [159].

#### 4.4.2. Time of Fertilizer Application

The time of fertilizer application is in tight connection with the amount of fertilizer application from the perspective of reducing N_2_O emission. Fertilizer application weeks after sowing instead of prior to sowing increases the chances that applied N will end up in crop tissues instead of getting lost to atmosphere and ground water. For instance, in maize, the side dressing of N at V-6 stage increased NUE and reduced N losses in the form of N_2_O [160,161]. Contrarily to this, the autumn application of fertilizers or manure enhanced nitrate and N_2_O losses [162,163].

**Table 1:** Effect of different sources of N fertilizers on N_2_O emissions.

Crop	N Sources	N_2_O Emission (kg ha^−1^)	References
Rice	Control (no fertilizers)	0.04	[164]
	AS (100 kg ha^−1^)	0.17	
	Urea (100 kg ha^−1^)	0.15	
Rice	Control (no fertilizers)	0.67	[116]
	NPK (210:105:240 kg ha^−1^)	6.51	
Rice	Control (no fertilizers)	0.64	[165]
	Urea (300 kg ha^−1^)	1.39	
Maize	Control (no fertilizers)	1.53 (kg N Mg^−1^)	[77]
	UAN (150 kg ha^−1^)	1.92 (kg N Mg^−1^)	
	CAN (150 kg ha^−1^)	1.81 (kg N Mg^−1^)	
Maize	Control (no fertilizers)	0.16	[166]
	Urea (145 kg ha^−1^)	0.30	
	AN (145 kg ha^−1^)	0.29	

UAN: Urea-ammonium nitrate, AS: Ammonium sulfate, CAN: calcium ammonium nitrate, AN: Ammonium nitrate, NPK: Nitrogen, phosphorus and potassium fertilizer.

#### 4.4.3. Improving N Fertilizer Placement

The deep placement of N fertilizers compared to conventional application ensures effective nutrient availability at later growth stages [167]. The placement of N closer to the plants considerably decreases N_2_O emission, as in the case of urea band application instead of broadcasting. Similarly, the side banding in wheat and canola, rather than the banded mid-row, appreciably reduced N_2_O emission [168]. In another study, the deeper placement of N fertilizer in maize resulted in a reduction in N_2_O emission compared to the shallow placement [168]. The site-specific N application according to field variability improves NUE by tailoring the applied N to soil spatial variability. In maize, site-specifically applied N reduced the overall N use by 25 kg/ha and resulted in a substantial reduction in N_2_O emission [169].

Deep placement of fertilizers is potentially useful to reduce N_2_O emissions [170]. In lowland rice [171], the deep placement of N fertilizers determined an 80% lower N_2_O emission than the conventional surface spreading. In another rice study [172], deep N placement substantially reduced N_2_O emission, owing to the fact that a large portion of N was retained in soil for a longer time. Moreover, Chapuis-Lardy et al. [173] argued that deep placement reduces N_2_O emission as a result of microbial consumption of N_2_O. Rutkowska et al. [174] also noticed a substantial reduction in soil N_2_O emission from sandy soils with deep placement of N fertilizers. Conversely, some other authors noted no significant difference in N_2_O emission with deep vs. broadcast application of fertilizers [175], and some others noted that deep placement of N fertilizers led to higher N_2_O emission [176]. It is sensed that these variations in N_2_O emission with deep placement vs. shallow placement or surface spreading can be attributed to differences in N source, the applied amount and interactions amid the soil and weather conditions [11].

#### 4.4.4. Selection of Suitable Fertilizers

Fertilizer type can influence N_2_O emission (Table 1) in association with time and amount of fertilizer application [177]. Fertilizers affect N_2_O emission because of different content of NH_4_^+^, NO_3_^−^ and organic C. Grave et al. [178] studied the impact of various N sources on N_2_O emission in a maize–wheat rotation. They noted that urea and slurry application increased N_2_O emission by 33% and 46%, respectively, as compared to the control plots. Bordoloi et al. [179] studied the impact of different levels of urea on N_2_O emissions in a wheat cropping system and found that N_2_O emission increased in parallel with urea increase, up to +174% N_2_O emission with 100 kg N ha^−1^ from urea. Moreover, Lebender et al. [180] studied the impact of N source (calcium-ammonium-nitrate (CAN; range 0–400 kg ha^−1^)) on N_2_O emission from the wheat crop. They noted that over the years, N_2_O emission from 400 kg N ha^−1^ was significantly higher as compared to 200 kg N ha^−1^. 

The experimental results reported in Table 1 clearly show the differences among fertilizer sources for N_2_O emission. Large differences can be seen among fertilizer forms [172,181]. Specifically, higher N_2_O fluxes and losses occur more quickly from ammonium nitrate compared to urea [182,183]. The application of calcium ammonium nitrate, especially in wet soils with high OM, results in higher N_2_O emissions [184]. In another work, Nayak et al. [185] reported that replacing urea with ammonium sulphate increases the N_2_O and decreases the CH_4_ emissions. However, further differences among N fertilizers for N_2_O emission can be due to soil properties, such as texture, BD, pH, organic carbon, N and microbial population [186].

Overall, fertilizer management is the premier domain of intervention to mitigate N_2_O emissions, as N fertilizers supply the nutrient that, to a varying degree (1.25% on average, according to the IPCC [153]), fuels N_2_O emission from agricultural soils. However, N fertilizers are a powerful tool to boost agricultural productions and are, therefore, indispensable to the present level of world food production. More efficient ways of supplying this nutrient, i.e., determining the right amount, time and place of supply, are the only strategy to pursue the increase in agricultural production necessitated by a growing population, while concurrently restraining N_2_O emission. Time and place of N application are the least controversial fields to achieve a significant containment of N_2_O emission at no cost to potential yield. The higher level of N application significantly increased N_2_O emissions [187,188]. The application of higher levels of N significant increases the DNF, which, resultantly, increases N_2_O emissions. Moreover, fertilizers and type of N also influence NF and DNF and, resultantly, N_2_O emissions. For instance, the application of anhydrous ammonia significantly increased N_2_O emissions [189]. Environmental conditions also significantly affect N_2_O emissions. The application of heavy doses of N can increase N_2_O emissions in warm temperate regions due to favorable microbial activities [190]. The tropical and sub-tropical zones also favor the microbial NF and DNF, which are linked to CO_2_ and N_2_O emissions [191] (Xu et al., 2012). Therefore, the application of heavy doses of N must be avoided in these regions. Moreover, Muller et al. (2003) [192] also observed N_2_O emission observed between −1 and 10 °C, and maximum N_2_O emissions occurred near the 0 °C owing to increasing activity of N_2_O reductase.

### 4.5. Biochar Application

**Figure 3:**
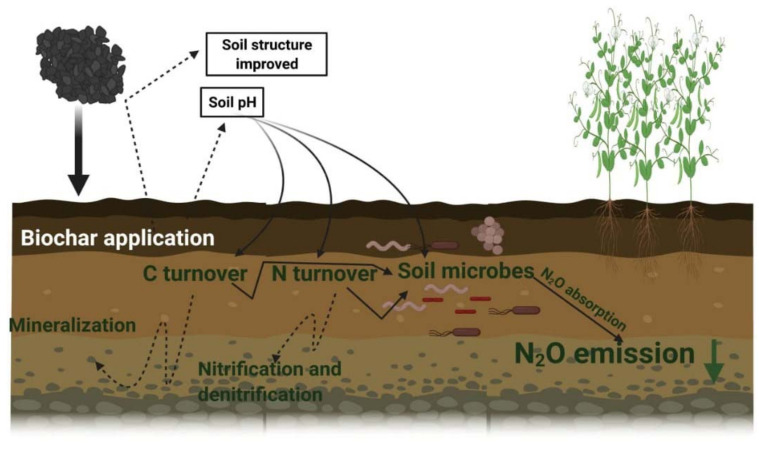
Mechanisms related to the role of biochar in mitigating N_2_O emission.

Biochar is a C-rich product resulting from the pyrolysis of various sources of organic matter. Soil incorporation of biochar sequesters C and improves soil properties [41,193,194], involving physical, chemical and biochemical changes (Figure 3), influencing N_2_O production [195]. The application of biochar can mitigate GHGs emissions from soils [196]. Because of slow degradation, biochar is considered as the best option for long-term carbon seizure in soils [197]. Biochar produced from plant biomass has a significant quantity of carbon that can be sequestered for up to 2000 years of mean residence time in soil [198]. Biochar application hinders GHGs emissions, therefore reducing global warming [197]. The application of biochar could reduce the emission of N_2_O and NH_3_ by 16.10% and 89.60%, respectively, as compared to control in rice crop [199].

The application of biochar increases soil pH and drives N_2_O complete reduction to N_2_, thus curbing N_2_O emission (Table 2) [200]. However, the impact of biochar on N_2_O emission varies according to biochar amount and soil properties, including pH, C:N ratio, organic carbon, water status, microbial and enzymatic activities. The biochar-mediated reduction in N_2_O emission is made possible by biotic and abiotic pathways [53]. The main effects of biochar, modification of soil pH, aeration and water-holding capability, are those responsible for reduced N_2_O emission [201]. However, biochar also directly absorbs N_2_O, which further contributes to reduced emission [202].

An enzyme, N_2_OR, catalyzes N_2_O transformation into N_2_ during the DNF. Under low soil pH, the assembly and functioning of this enzyme are constrained [202]; the application of biochar, by increasing soil pH, restores N_2_OR functioning, which explains the relevant reduction in N_2_O emission following biochar application [203]. The increase in aeration and O_2_ availability resulting from biochar application contributes to further reduction in N_2_O emissions by creating adverse conditions for microbial DNF [203].

**Table 2:** Effect of biochar on N_2_O mitigation potential compared to no biochar application.

Biochar Application	N_2_O Mitigation Potential (%)	Reference
BBC: 5 tons/ha	38	[204]
BBC: 10 tons/ha	48	
BBC: 15 tons/ha	61	
RCHBC: 50 tons/ha	36	[205]
MSBC: 16.77 tons/ha	10.8	[206]
BBC: 5 tons/ha	24.25	[207]
BBC: 15 tons/ha	30.7	
RSBC: 22.4 tons/ha	72.95	[208]
RSBC: 44.8 tons/ha	235.1	
RSBC: 36 tons/ha	50	[209]
RSBC: 72 tons/ha	83	
WSBC: 10 tons/ha	101.68	[210]
CSBC: 9 tons/ha	46.3	[211]
CSBC:13 tons/ha	33.3	
RSBC: 1% (*w*/*w*)	82.28	[212]
RSBC: 5% (*w*/*w*)	185.21	

GHBC: Grain husk biochar, BBC: Bamboo biochar, RCHBC: Rice and cotton husk biochar, MSBC: Maize stalk biochar, RSBC: Rice straw biochar, WSBC: Wood shaving biochar, CSBC: Cotton stalk biochar.

Additionally, biochar has a good adsorption potential, resulting in a considerable adsorption on its surface of NH_4_^+^ and NO_3_^−^ [213], which reduces the N availability for N_2_O production [214]. Biochar application also influences soil gene abundance, including nirK and nosZ [215]. These genes are highly sensitive to acidic pH, and they are involved in the process of DNF. The nosZ gene is linked to N_2_O reductase, which catalyzes the reduction of N_2_O to N_2_ [216]. This is a further reason for biochar application resulting in substantial reduction in N_2_O emission [217,218]. The application of biochar not only increases the SOC, crop yield and soil fertility, but also influences N_2_O emissions. Many authors noted that biochar application reduced N_2_O emission from agricultural soils [199]. However, environmental and soil conditions are significant factors that affect N_2_O emissions. A meta-analysis conducted by Shakoor et al. [219] showed that application of biochar to fine textured soils significantly increased N_2_O and CO_2_ emissions. However, biochar application to coarse textured soils had no impact or reduced N_2_O [219]. Under all circumstances, these effects can be best predicted by soil moisture and environmental conditions. Therefore, biochar application as a long-term approach to reduce N_2_O emission appears quite promising, owing to the fact that the literature does not report any controversy in biochar’s final effects. However, detailed mechanisms need to be further elucidated in order to assure higher reliability and, therefore, profitability of this practice.

### 4.6. Lime Application 

Lime application modifies soil pH, which regulates different soil processes, including OM mineralization, NF and DNF, which in turn affect soil N_2_O production [57,220]. However, contradictory reports have been issued regarding the impact of lime on N_2_O emission, as the increased C and N mineralization, the latter resulting in higher NH_4_^+^ and NO_3_^−^ contents, are the premise for enhanced NF and DNF, potentially leading to N_2_O emission [52,200]. Conversely, other studies pointed out a significant reduction in N_2_O with lime application, thanks to the increased N_2_O reductase activity, resulting in more N_2_ in exchange for less N_2_O as the ultimate reduction product [221,222,223].

Soil N_2_O emission is regulated by pH; N_2_O emission decreases linearly with increased pH in a pH range of 4–7, irrespective of soil type [224]. The liming material also has great impact on the mineral N content. The addition of lime reduces NH_4_^+^ and speeds up the NF process, increasing NO_3_^−^ content. The higher NO_3_^−^ content at high pH stimulates micro-organisms to consume N_2_O as electron acceptor in lieu of NO_3_^−^ [225]. Thus, lime potentially ensures the complete DNF and promotes N_2_O conversion to N_2_. The increase in dissolved organic carbon associated with liming serves as a readily available C source for microbial growth, further contributing to N_2_O abatement [226].

It is, therefore, evinced that liming acidic soils has an intrinsically favorable role in containing N_2_O emissions; yet, the increase in readily available N forms, namely nitrates, is a potential source of N_2_O, which deserves to be directed toward plant nutrition in the first instance or needs to be ultimately denitrified to N_2_ in the second instance. In other words, the undeniable benefits of liming need to be carefully exploited in order to limit NO_3_^−^ residual amounts which, under unfavorable conditions, fuel N_2_O emission.

### 4.7. Use of Nitrification Inhibitors or Slow-Release Fertilizers

Nitrification inhibitors (NI) or slow-release N fertilizers can reduce both N_2_O and CH_4_ emissions [227]. The NI reduces N_2_O emission directly, by inhibiting NF, as well as indirectly, by reducing NO_3_^−^ availability for DNF [228], without compromising yield [229,230]. The chemical compounds present in the NI deactivate the enzymes responsible for the first step of NF (ammonia mono-oxygenase; AMO), maintaining NH_4_^+^ for longer periods in soils [231,232]. As a result, the NI decreases the rates of NF and the availability of substrates for denitrifiers, in turn reducing N_2_O emission from fertilizers [233]. Various authors noticed a significant reduction in N_2_O emission with application of different NI, including dicyandiamide, hydroquinol, nitropyrimidine and benzoic acid [234,235]. Lastly, plant-derived products, such as neem oil, neem cakes and karanja seed extract, can be used to inhibit NF; however, the exact mechanisms behind NF reduction induced by these products are still unclear.

The quest for NUE improvement is oriented toward the utilization of slow-release fertilizers, in order to reduce N_2_O emission and the effects of global warming [94]. Slow-release fertilizers are mainly represented by controlled-release fertilizers (CRF) [236]. The CRF are granule-coated fertilizers, which slowly release the nutrients in order to improve nutrient uptake efficiency [237], reducing N losses by delaying the initial N supply and gradually providing the nutrient to the plants [238]. The application of CRF is recommended for those areas where the vulnerability to N losses is very high [239]. In paddy rice, the application of CRF significantly reduced N_2_O losses and N application rate by 26–50%, without compromising yield [240]. The application of CRF can be seen as an effective approach to mitigate the N losses in combination [241] or as an alternative to urea [242]. 

It may be concluded that NI and CRF application is a promising approach to curb N_2_O emission and other pathways of N loss, while concurrently improving crop production and NUE. The gradual release of nitrogen determined by both types of products ensures no peak of N supply responsible for increased N_2_O emission. The main constraint in the use of NI and CRF is represented by their cost, which needs to be carefully evaluated in view of the expected return.

### 4.8. Use of Organic Amendments 

Organic amendments (OA), including CR and animal wastes (i.e., manures and slurries), have been widely used to reduce N fertilizer application, improve soil fertility and alleviate environmental deterioration [3,14,243,244]. The effects of OA on N_2_O emission have been documented in both lab and field studies. Some researchers demonstrated that OA enhance N_2_O emission through DNF by serving as energy source for denitrifiers, favoring the formation of anaerobic micro-sites within soil aggregates [245,246]. Conversely, other researchers showed that OA reduce N_2_O emission by increasing N microbial assimilation, thus limiting the availability of N substrates for the production of N_2_O through NF and DNF [247,248]. The difference between these two contrasting behaviors could be due to differences in OA application, soil and climatic conditions, and fertilization history in the respective studies [249,250]. A long-term study showed that the amount of OA is critical for the accumulation of organic carbon and subsequent impact on N_2_O emission [251]. Moreover, it is assumed that the substitution ratio of synthetic fertilizers by OA is an important feature regulating N_2_O emissions [251]. 

Therefore, OA are a viable alternative to mineral N fertilizers, in whose respect they do not provide clear advantages, as OA denote potential benefits as well as drawbacks in terms of N_2_O emissions, depending on specific cases. Based on this, it is not easy to trace a consistent behavior for N_2_O abatement through OA; it may only be concluded that a sensitive use of OA can contribute to an alleviation of the N_2_O problem, whereas an unconsidered use of OA may result in aggravating the N_2_O problem. Generally, NF is considered to be a major source of N_2_O emission under limited moisture conditions; however, optimum moisture conditions in irrigated soils can induce anaerobic conditions, which promote the DNF [132]. Manure application ensures quick availability of C-substrates that promote the activity of DNF bacteria and increase the development of micro-sites due to higher moisture contents, which promote N_2_O production and emissions [252,253]. Therefore, the application of organic manures in areas with higher rainfall and the application of heavy irrigation could increase N_2_O emissions as compared to dry areas. 

### 4.9. Fermented Organic Manures

The incorporation of fermented manures to soil can reduce GHG emission owing to rapid depletion of the pools of OM during fermentation [254]. The application of fermented CR significantly reduced CH_4_ emission by 52% compared to application of fresh residues in a lab experiment [255]. A huge difference has been documented among GHG emissions triggered by fresh and pre-fermented materials [256]. For instance, the application of fermented biogas residues increased the CH_4_ emission by 42%, while the unfermented material increased the CH_4_ emission by more than 110% [234]. In another investigation, Nayak et al. [185] found that composted manure application significantly decreased N_2_O and increased C sequestration and CH_4_ emission. In rice, Zhang et al. [76] reported that compost application reduced N_2_O emission by more than 50% compared to urea. The application of organic material produced as a result of aerobic composting of rice straw considerably reduced GHG emissions (CH_4_ and N_2_O) compared to fresh straw [255], suggesting that this approach is environmentally friendly. 

It appears, therefore, that OA obtained from organic matter fermentation do not show harmful effects in the literature, possibly in association with more controlled doses with respect to OA originating from animal slurries and manures. Higher N_2_O emissions in manure-amended and irrigated soils are a major concern in the climate-resilient agroecosystems [132] (Shakoor et al., 2022). Generally, the application of manures to irrigated lands increases N_2_O emissions due to substrate availability and increasing micro-sites and microbial activities [252,253]. Higher rainfalls can also induce a significant increase in N_2_O emissions following the application of fermented manures. Therefore, it could be suggested that manure application in irrigated soils and areas facing higher rainfall be dealt with cautiously to ensure better production and lower N_2_O emissions. 

### 4.10. Composting

Fermentation refers to a breakdown of organic substances into energy and by-products under anaerobic conditions, whereas composting involves the degradation of organic materials into value-added products under aerobic conditions. The application of composted materials has been widely practiced in crop production [6,256,257,258]. The dissolved organic carbon (DOC) released from composted animal manures can be a source of available C for microbial use in DNF, and the cumulative N_2_O emission is directly related to the concentration of DOC in soil [72]. Vermi-composting is a promising approach that involves the conversion of organic materials into compost in the presence of earthworms [208,259]. The material produced as result of their activity has good structure and microbial activity associated with the abundance of liable resources. In a study on rice, the application of vermi-compost decreased the transfer of NH_4_^+^ and NO_3_^−^ to water [260].

However, extensive use of vermi-compost might increase N_2_O gaseous losses, owing to higher N availability, stimulating microbial activity. In fact, the combined use of vermi-compost and inorganic fertilizers increased N_2_O emission by increasing the NO_3_^−^ concentration with respect to unamended soil [261]. Conversely, the combined application of biochar and vermi-compost influenced soil properties through the C:N ratio and by increasing the abundance of *nosZ* genes; all of this led to reduced N_2_O emission [262]. Therefore, the combined application of biochar and vermi-compost may be a promising approach to reduce N_2_O emission. However, more studies are needed on a large scale to determine the influence and interaction of biochar and vermi-compost on N_2_O emission and the mechanisms lying behind the reduction in N_2_O emission due to these products. 

Therefore, as in the case of fermented manures, the application of composted materials appears to be a promising strategy to improve soil properties and the general fertility. This, in turn, will likely result in restrained N_2_O emission.

### 4.11. Role of Arbuscular Mycorrhizal Fungi

The understanding of the N_2_O production pathway has been significantly improved recently by the development of isotopic methods for tracing the sources of N_2_O [263,264]. N_2_O production rate from soils is controlled by the available N, soil pH, OC, N, microbial activity and oxygen availability [26,265]. Arbuscular mycorrhizal fungi (AMF) are a key group of micro-organisms that form symbiotic relationships with most plants [38,39]. It is generally acknowledged that AMF play a role in the N cycle, as they can acquire this nutrient for host plants and have N requirements for themselves [39,40,266]. It has also been documented that AMF reduce NO_3_^−^ leaching [267,268]. In general, these fungi reduce the availability of N sources in NF and DNF for the production of N_2_O. AMF are able to acquire both NH_4_^+^ and NO_3_^−^; nonetheless, they prefer the more energetically attractive NH_4_^+^ [38,39,269]. The competition of these fungi with other micro-organisms for inorganic N reduces the N availability for N_2_O producers and the consequential N_2_O emission [270]. Another study highlighted a significant reduction in N_2_O emission from soils affected by AMF-colonized roots compared to soils influenced only by root activity [271]. Similarly, another research outlined a reduction in N_2_O fluxes in the rice crop by means of AMF [272]. The above-mentioned studies suggest that AMF alter N_2_O emission; however, it has not been determined whether AMF induce N_2_O reduction by physiological changes in the AMF-colonized roots or as direct result of the AMF themselves. Recently, it has been noticed that AMF directly reduce N_2_O emission [249]. Additionally, AMF also affect the N cycling by capturing the nutrient and transferring some portions to host plants [273]. The availability of N and C are the factors that control NF and DNF [274]. Thus, it is not possible to separate the AMF and root fluxes of N_2_O in the mycorrhizosphere without first separating the AMF hyphae from plant roots. Additionally, there is a positive association between the presence of AMF and reduced NF [38,39]. Likewise, the presence of AMF reduces the abundance of *nirk* genes, which are considered responsible for N_2_O production [274] Thus, a decrease in N_2_O in the presence of AMF can be due to lower NF rates [274]. Additionally, AMF reduce NH_4_^+^ in the hyphosphere, resulting in a reduction in ammonia-oxidizing bacteria (AOB) population. Since AOB are considered the main producers of N_2_O, this may explain the reduction in N_2_O emissions owing to AMF activity [274].

It is definitely evinced that AMF, by interacting with the host plant and the soil environment, can play a relevant role in restraining N_2_O emission. Specifically, AMF activity buffers the content of available N forms in soil profile, which in turn results in lower amounts of NO_3_^−^ prone to DNF. All the consulted sources are consistent with a potentially beneficial role exerted by AMF in restraining N_2_O emission.

### 4.12. Selection of Plant Genotypes

The selection of suitable cultivars is a prerequisite to obtain the desirable crop production [275,276,277,278,279,280], while concurrently playing a role in GHG reduction. The variations amid the rice cultivars for CH_4_ emission can be related to differences in CH_4_ production, oxidation and transport [281]. The mechanisms explaining the differences among plant species for N_2_O emission are often unclear [282]; however, numerous prospects can be envisioned. In the case of the rice plant, active pathways exist for N_2_O transport through aerenchyma cells to soil submerged with water [283], and during daytime, N_2_O is transported from roots to shoots via the transpiration stream and is subsequently lost through stomata [284]. In *Brachiaria humidicola*, a tropical grass, there are cultivars able to produce the chemicals that directly inhibit NF [285], substantially reducing N_2_O emission [286]. In another study, it was noticed that the lowest N_2_O emission was linked with a plant strategy characterized by higher N uptake [287]. In fact, plant cultivars with higher N uptake were shown able to reduce the N pool, especially NO_3_^−^, resulting in lower availability of substrate for denitrifiers and subsequently lower N_2_O emission. The variation amid cultivars for N_2_O emission had also been reported in the intercropping of cereals and legumes [288]. In another study, researchers noticed a significant contribution of plants to N_2_O emission and suggested that in the soil-crop system, N_2_O emission is markedly influenced by plant characteristics [289]. 

Therefore, it appears that the breeding of crop plants could be directed, among other things, to the release of cultivars, enabling N_2_O containment. All plant strategies conducive to earlier and stronger N uptake deplete soil reserves and leave less NO_3_^−^ exposed to the risk of N_2_O production. In this respect, a relevant goal from a productive viewpoint can be associated with breeding with an equally relevant goal from an environmental viewpoint.

### 4.13. Modifying Cropping Schemes and Crop Rotations 

In rice, switching from conventional puddled transplanted rice (TPR) system to directly seeded rice (DSR) may contribute to reducing GHG emissions. In fact, it was noticed that DSR increased N_2_O emission when the redox potential (RP) crossed 250 mV [290]. It was concluded that water should be applied in such a way that RP be kept at a range of 100–200 mV to reduce both N_2_O and CH_4_ emissions. Since DSR system offsets N_2_O emission, it is an encouraging production system, thanks to the lower GWP [230]. The DSR has 53% less GWP in terms of N_2_O, CH_4_ and CO_2_ components as compared to traditional TSP [291]. Further, Ahmad et al. [112] stated that GWP of DSR can be further decreased by shifting toward no tillage (NT). The lower GWP and higher production of DSR suggest that DSR would decrease both CH_4_ and N_2_O emissions. Nonetheless, more detailed studies involving the measurements of GHGs under the concurring effects of factors including water, tillage, nutrients and biochar, are direly needed to support DSR as a suitable system that also reduces the environmental burden.

Few studies investigated the impact of crop rotation diversity on GHG emissions from diverse plant species within the rotation. The GHG fluxes were investigated under a maize–soybean rotation for three years, and it was noticed that maize and soybean emitted a similar amount of CH_4_ [292]. In another study, authors reported non-significant differences in N_2_O emission from different species, including cowpea, wheat and soybean, in a four-year rotation [293]. In some other works, the authors compared N_2_O emissions from crops sown in rotation and mono-cropping; corn sown in rotation decreased the N_2_O and CO_2_ emissions compared with continuous corn [294,295], owing to the application of large amount of N fertilizers in mono-cropping. However, some authors noticed that wheat grown in rotation and in mono-cropping emitted the same amount of N_2_O [296]. In another study, maize staged the same N_2_O emissions when grown as continuous crop and in maize–soybean and soybean–wheat–maize rotations [297]. Crops entering a cropping system must be chosen properly because they significantly affect N_2_O emissions [219]. For instance, grasslands significantly increased N_2_O emissions, whereas maize crop showed a negative impact on N_2_O emissions [219]. Intensive grasslands can increase global N_2_O emissions owing to the application of manures and animal excreta deposited on the surface of grasslands [298].

Such differences suggest that the effect of crop rotation diversity on GHG emission can vary owing to soil and climate conditions, and crop diversity. Since there is no univocal effect exerted by cropping schemes and rotations, the amount of N_2_O emissions and their potential abatement appear to be linked to specific issues in crop management, such as the planting system or N fertilization, whose effects have already been surveyed in the specific sub-sections.

### 4.14. Integrated Nutrient Management 

Integrated nutrient management (INM) involves the combined use of OA and inorganic fertilizers to increase NUE and reduce N losses by synchronizing crop demand with soil nutrient availability [35,299]. A few reports are available about the effects of INM on GHG emission. Some authors compared the effects of NPK fertilizer, compost and their combination on N_2_O emission [299,300]. They noted that a combined application of NPK and compost reduced N_2_O emission compared to the sole use of compost or NPK. Additionally, they suggested that the application of composted material with C:N ratio lower than 20 significantly reduced N_2_O emission, owing to the release of a lower amount of N during decomposition in soil [299,300]. Moreover, one research work measured the impact of INM (cattle manure and AN) on N_2_O emission during one growing season for maize and wheat. These authors noted that INM increased N_2_O emission compared to cattle manure, whereas it decreased the emission compared to AN. This reduction in N_2_O emission with the application of OA was due to slower decomposition of C and N, and slower release of mineralized N [301]. Huang et al. [59] noticed the reduction in N_2_O emission with plant amendments at increasing C:N ratio and found that this relation becomes stronger with the addition of inorganic N. Nonetheless, in this study, the treatment featuring highest N_2_O emission was associated with the greatest N supply, indicating that the N dose effect remains of paramount importance. In accordance with the previous results, another study suggested that a reduction in N_2_O emission occurs when OA with lower C:N ratio are applied alone or when OA with higher C:N ratio are applied together with inorganic fertilizers [302].

Nonetheless, rare field studies are available about the effect of the C:N ratio of OA on N_2_O emission. As the micro-organisms involved in the NF and NDF processes depend on C supply, the application of OA with a C:N > 20 tends, under no synthetic N supply, to result in nutrient microbial immobilization, in turn reducing the available N for DNF [303]. Conversely, OA with lower C:N ratio are more quickly mineralized by microbial activity and result in the release of C and N, which increases the microbial activities and, resultantly, N_2_O emission [303]. Nonetheless, the microbially induced N_2_O emission from INM not only depends on C:N ratio but also on the amount of synthetic N fertilizers added to soil. The application of N fertilizers with OA with a large quantity of labile C further increases DNF, leading to higher N_2_O emission [304]. A summary of studies indicates that the INM leads to a reduction in N_2_O emission (Table 3).

The total rate of N applied from OA and inorganic fertilizers also explains the amount of N_2_O emission [300,307]. It is not surprising that the INM approach of combining organic and synthetic N sources at higher N rates results in higher N_2_O emission compared to their alternative use at lower N rates [308]. Conversely, when half of the suitable N rate was applied from organic and half from inorganic sources, this resulted in reduction in N_2_O emission compared to the sole application of organic or inorganic N sources at the same N rate [308,309,310]. It is evinced, therefore, that combining OA with inorganic fertilizers does not assure reduction in N_2_O emission. However, a meta-analysis conducted by Graham et al. [295] suggests that the application of amendments with very low C:N (<8) ratio in a substitutive strategy of N application (proportional reduction in N rate from each N source) has a good potential to mitigate N_2_O emission. Therefore, the integrated use of inorganic N with OA at lower C:N ratio helps to avoid two processes, namely rapid mineralization of inorganic N (low C:N ratio) and stimulation of microbial activity through the addition of excessively C-rich substrates (high C:N), which together contribute to N_2_O emission. 

It is perceived, in general, that only a shrewd application of INM can make this approach successful in the quest for mitigating N_2_O emission. It is equally sensed that none of the crop practices previously surveyed, nor INM alone, can positively contribute to alleviating this problem, unless N_2_O abatement is considered a major goal in crop production and practices in crop management are directed toward its achievement.

## 5. Role of Regulatory Authorities in Implementing Environment-Friendly Management Practices to Reduce GHGs Emissions 

The intensity of GHGs has substantially increased in recent time, which has in turn increased climate change and global warming [311,312,313,314,315,316]. Globally, various policies, measures and strategies are being deployed by governments to limit GHGs emissions. Different approaches, including standards, incentives and different permissions, are used to encourage environmentally friendly approaches to restrict GHGs emissions [317,318]. However, these approaches may vary at the national and sub-national levels according to each country. GHGs are major drivers of climate change, and diverse international negotiations have taken place in the last two decades to curb GHG emissions and counter climate change and global warming. Many countries have followed various development cycles since the 1990s to reduce GHGs emission. Initial efforts were made in reducing GHG emissions from developed and industrialized nations, which eventually became the Annex-1 group of the Kyoto Protocol [319]. Similarly, the 27 member states of the European Union (EU-27) and the United Kingdom have signed commitments to become carbon-neutral economies by the end of 2050 [320]. Moreover, the European Commission also proposes to reduce GHG emission by 55% compared to 1991 by the end of year 2030 [321,322]. However, the simultaneous implementation of climate change policies in the EU-27, UK and USA has also put a major focus on heavy industries as the main source of national gross domestic product [323]. By contrast, some medium to large countries have also gone through unprecedented economic growth as a result of industrialization, and they are also experiencing a substantial increase in population growth [324]. The socio-economic and demographic transformations combined with technology are designed to restrict climate change and GHG emissions in a framework of market conditions. An important practice adopted around the globe is the use of renewable energy sources accompanied by the decrease in use of coal and petroleum and the development of efficient energy production and consumption practices [325,326]. 

During the 1997 UNFCC conference of parties in Kyoto, a protocol was adopted, and it was enforced in 2005. This Kyoto Protocol invented the GHG emission commitments for developed nations for a period of five years (2008–2012). The Kyoto Protocol defined four emission-saving units, including those obtained: (1) by clean development mechanism projects, (2) through joint implementation of projects, (3) through the trading of unused assigned emissions between protocol parties and (4) through reforestation-related projects. Moreover, during the year 2012, an amendment was made to the Kyoto Protocol, and a second commitment period was determined for another seven years (2013–2020) to reduce GHG emission. The proposed amendment targeted a reduction of 18% in GHG emission as compared to 1990 levels [327]. 

Nowadays, it has been recognized that environmental protection is an essential part of business processes [328]. Environmental protection can yield many benefits, including cost and resource savings, and it can increase satisfaction and loyalty in people [329]. The European Commission developed the European Union (EU) Eco-Management and Audit Scheme (EMAS) for companies and other sectors to adopt the environmentally friendly approaches to restrict environmental footprint [330]. The Environmental Management Systems (EMS), such as ISO (International Organization for Standardization) or EMAS (Eco-Management and Audit Scheme), have been also designed for ensuring higher environmental protection and competitive advantage of organizations resulting from the introduced improvements. Corporate social responsibility (CSR) is another important concept in performing business activities according to which companies still make a profit in strict compliance with the law, and they take into account the impact of their operations on the environment in their business decisions [328]. The application of such approaches improves the quality of life and ensures a sustainable development. 

## 6. Conclusions and Future Prospects 

The mushrooming population and rapidly increasing food demand have raised the concern all around the globe of stabilizing the atmospheric greenhouse gases concentration for mitigating the ongoing climate change. Here, we presented comprehensive information about management practices designed to reduce N_2_O emission.

The adoption of all the practices reviewed here is expected to mitigate N_2_O emission without comprising productivity. The discussion of the literature allowed us to outline the role of management options that can be adopted either alone or in association, in the quest to reduce N_2_O emission. Prioritizing the use of fertilizers associated with low N_2_O emission, such as ammonium fertilizers, leads to less N_2_O compared to nitrate fertilizers. Similarly, the deep placement of N fertilizers should be promoted to reduce N_2_O emissions. Plant-breeding activities should be aimed at releasing genotypes with better N uptake, nitrogen fixation and the ability to capitalize those C–N interactions in the rhizosphere, which can be helpful to reduce N_2_O emission. Promoting sustainable crop intensification, which can be done by using higher-yielding crop varieties, reducing the use of external inputs, improving nitrogen use efficiency, using biochar and lime to counter acidic soil pH and adopting agroecological practices, can help to mitigate the impact of current management systems on N_2_O emissions. The selection of suitable irrigation methods is an important strategy to save water and maintain yields. However, future studies are needed to study irrigation effects on soil hydraulic properties, which affect water distribution and, therefore, N_2_O emission. Additionally, these systems are often combined with fertilizer applications, thus future work is required to evaluate the impact of rate, frequency and types of N fertilizer on N_2_O emission under sprinkler and drip irrigation systems.

Moreover, to further understand the impact of C:N ratio on N_2_O emission, integrated nutrient management studies should be conducted by including a wider range of C:N ratios in organic amendments, along with the application of inorganic fertilizers. In parallel to this, different organic amendments with similar C:N ratio should be applied with constant rates of nitrogen to better appraise the impacts of organic amendment properties beside C:N ratio on N_2_O emission. In arbuscular mycorrhizal fungi, future studies should be conducted to explore their interaction with microbial communities, including ammonia-oxidizing archaea and bacteria, nitrifying communities and non-denitrifying N_2_O reducers.

A better understanding of successful N_2_O mitigation strategies requires studies related to N_2_O fluxes in agroecosystems to account for the wide range of biotic and abiotic factors, including ecosystem state factors, such as soil characteristics, climate and topography, which interact with management practices to influence soil N_2_O emission. Nonetheless, only few of the above-mentioned studies consider the interactions between eco-system state factors and management practices. Therefore, interdisciplinary and cross scale studies should be run to understand how we can successfully reduce N_2_O emission in crop production systems. Finally, at the field level, N_2_O measurements and agronomic information can be used to design N_2_O mitigation approaches that should reduce the carbon footprint and maximize monetary paybacks of cultivation efforts.

## Figures and Tables

**Table 3:** Effect of organic/inorganic nutrients and integrated nutrient management (INM) on N_2_O emission.

Crop Rotation	Total Rate of N (kg ha^−1^)	N_2_O Emission Trend	References
Maize–wheat	Organic: 150 composted manure (CM), Inorganic: 150 urea, INM: 75 CM + 75 Urea	No significant difference was recorded	[300]
Maize–wheat	Organic: 150 CM, Inorganic: urea, INM: 75 CM + 75 urea	No significant difference was recorded	[301]
Maize–wheat	Organic: 150 CM, Inorganic: urea, INM: 75 CM + 75 urea	INM, Organic < Inorganic	[302]
Rapeseed	Organic: 97.5 cattle manure, Inorganic: ammonium nitrate (AN) 120, INM: 65 cattle manure + 60 AN	INM < Organic, Inorganic	[303]
Maize–wheat	Organic: 120 cattle manure, Inorganic: AN 120, INM: 60 CM + 60 AN	Organic < INM < Inorganic	[304]
Maize–wheat	Inorganic: 100% NPK, INM: 100% NPK + FYM	Inorganic < INM	[305]
Rice	Inorganic: 120 kg urea, INM: Compost (30 kg/ha + urea 90 kg/ha	INM < Inorganic	[306]

## Data Availability

Not applicable.
